# Ethnic Differences in Survival Among Lung Cancer Patients: A Systematic Review

**DOI:** 10.1093/jncics/pkab062

**Published:** 2021-07-07

**Authors:** Sarah N Price, Melissa Flores, Heidi A Hamann, John M Ruiz

**Affiliations:** 1 Department of Psychology, University of Arizona, Tucson, AZ, USA; 2 Center for Border Health Disparities, University of Arizona Health Sciences, Tucson, AZ, USA; 3 Department of Family and Community Medicine, University of Arizona, Tucson, AZ, USA

## Abstract

**Background:**

Despite a substantially worse risk factor profile, Hispanics in the United States experience lower incidence of many diseases and longer survival than non-Hispanic Whites (NHWs), an epidemiological phenomenon known as the Hispanic Health Paradox (HHP). This systematic review evaluated the published longitudinal literature to address whether this pattern extends to lung cancer survival.

**Methods:**

Searches of Medline, PubMed, Embase, Web of Science, and the Cochrane Library were conducted for publications dated from January 1, 2000, to July 18, 2018. Records were restricted to articles written in English, employing a longitudinal design, and reporting a direct survival comparison (overall survival [OS], cancer-specific survival [CSS]) between NHW and Hispanic lung cancer patients.

**Results:**

A final sample of 29 full-text articles were included, with 28 fully adjusted models of OS and 21 of CSS included. Overall, 26 (92.9%) OS models and 20 (95.2%) CSS models documented either no difference (OS = 16, CSS = 11) or a Hispanic survival advantage (OS = 10, CSS = 9). Both larger studies and those including foreign-born Hispanics were more likely to show a Hispanic survival advantage, and 2 studies of exclusively no-smokers showed a survival disadvantage. A number of reporting gaps were identified including Hispanic background and sociodemographic characteristics.

**Conclusions:**

Hispanics exhibit similar or better survival in the context of lung cancer relative to NHWs despite a considerably worse risk factor profile. These findings support the HHP in the context of lung cancer. Further research is needed to understand the potential mechanisms of the HHP as it relates to lung cancer.

Despite improvements in screening, detection, and early intervention, lung cancer remains the leading cause of cancer death in the United States, currently accounting for more than 135 000 annual deaths (1). Both racial and socioeconomic status (SES) disparities are associated with lung cancer incidence and mortality; non-Hispanic Black (NHB) men, those without a high school degree, and those under the poverty level experience the greatest lung cancer burden and highest rates of death (2). A variety of factors, including smoking status, tumor biology, access to care, and treatment inequities, are demonstrated contributors to these robust racial and SES disparities (2,3).

There are clear connections between race, SES, and lung cancer disparities for NHBs, but these relationships are not as straightforward for Hispanics and Latinos (hereafter, referenced as Hispanics). Like NHBs, Hispanics tend to have lower SES and are more likely to be diagnosed with late-stage disease and to experience treatment delays (4,5). They also have substantial health-care barriers, including under- and uninsured status, lower quality of care, and lack of regular care providers (4-6). These care disparities, coupled with broader socioeconomic disadvantages, should predict incidence and survival disparities for Hispanics relative to non-Hispanic Whites (NHWs). However, as clearly demonstrated by incidence data, Hispanics are less likely to be diagnosed with lung cancer than NHWs (27.7 vs 56.1 per 100 000) (4). Although this difference may be partially driven by lower smoking rates among Hispanics compared with NHWs (10.7% vs 16.6%) (7), Hispanics still experience a lower risk of lung cancer when compared with NHWs with similar smoking histories (8,9). Furthermore, emerging evidence suggests that Hispanics have a survival advantage in the context of a lung cancer diagnosis. For example, a recent meta-analysis reported relative survival advantages for Hispanic lung cancer patients compared with NHWs (10). Although this study was limited in scope, including only 5 studies based on breadth of search (eg, PubMed exclusively) and study-specific inclusion criteria, it supports the possibility of a Hispanic survival advantage in the context of lung cancer.

A survival advantage in the context of lung cancer would be consistent with an epidemiological phenomenon commonly referred to as the Hispanic Health Paradox (HHP). The HHP is supported by the large body of research demonstrating lower incidence of most diseases and greater longevity in the context of disease than NHWs (11). For example, Hispanics experience lower rates of cardiovascular disease, many types of cancer, and stroke and live equally as long or longer in the context of these conditions compared with NHWs (12,13). These effects are large, robust, and replicated (11,14,15).

Using the Preferred Reporting Items for Systematic Reviews and Meta-Analyses (PRISMA) guidelines (16,17), the current aim was to extend the existing literature through a large-scale systematic review that more definitely addressed whether Hispanics have better lung cancer survival outcomes compared with NHWs. Based on risk profile disparities among Hispanics, studies were considered consistent with the HHP if they either documented Hispanic survival advantages or showed comparable survival outcomes between lung cancer groups.

## Methods

### Search Strategy

This systematic review was registered on the International Prospective Register of Systematic Reviews (PROSPERO; CRD42018115081). Preliminary searches revealed a paucity of available studies published before the 2000s; thus, January 2000 was used as the beginning search date based on this “natural window.” After consultation with a university medical librarian, we conducted electronic database searches for publications dated from January 1, 2000, to July 18, 2018, using Medline, PubMed, Embase, Web of Science, and the Cochrane Library. To capture the largest possible sample of potential articles, 2 search term categories were used: 1) Hispanic (Hispanic, Latino, Mexican, Puerto Rican, Cuban) and 2) lung cancer (lung cancer, thoracic neoplasm, non-small cell lung cancer [NSCLC], small-cell lung cancer [SCLC]) (see Supplementary Methods, available online, for example, search strategy). Searches were restricted to articles and abstracts with publication dates from 2000 to 2018, published in English, and involving human subjects. We then supplemented electronic searches by manually examining the reference sections of past reviews and studies meeting inclusion criteria to locate articles not identified in the database searches. Lastly, when information was incomplete, we contacted authors of potentially relevant abstracts to request additional papers or relevant data.

### Eligibility Criteria

The population of interest for this review was patients aged 18 years and older diagnosed with lung cancer, including any histological type or stage. We included only published studies meeting the following criteria: 1) written in English; 2) using a longitudinal design; 3) providing a direct survival comparison between NHWs and Hispanics with lung cancer, reported in terms of hazard ratios (HRs) or risk ratios (RRs). Primary outcomes included overall survival (OS) and lung cancer–specific survival or cause-specific survival (CSS), and articles were separated based on type of survival measured. Both OS and CSS outcomes were included as they offer distinct information relevant to patients, clinicians, and other stakeholders.

We included only studies with individual data and excluded reports with exclusively aggregated data (eg, census-level statistics). Randomized, controlled trials or longitudinal, nonexperimental observational studies were considered for inclusion. Conference abstracts were conditionally included in our initial search, but were only included in the final literature review if the study had been published in a refereed, full-text article. Authors of such works were contacted to verify whether a full-text article could be identified.

### Data Extraction and Quality Assessment

The results of the computerized search were imported into EndNote X8 by the first author (SNP). All abstracts were read to identify studies potentially meeting inclusion criteria. To identify eligible studies, 2 independent reviewers (SNP and MF) first screened titles and abstracts of all articles, and if the article could not be excluded based on the title and/or abstract, both reviewers then separately consulted the full text article to determine eligibility. After conducting independent reviews, SNP and MF compared results and resolved the majority of discrepancies. A third independent reviewer (JMR) then resolved any outstanding discrepancies through joint review and discussion with the primary reviewers. Final review inclusion was confirmed by consensus between all study authors.

Although the feasibility of a meta-analysis was explored, it was determined that such an analysis was not appropriate given the substantial overlap of data used (ie, Surveillance, Epidemiology, and End Results [SEER] Registry or California Cancer Registry) between studies and the inability to determine exactly which cases overlapped and had potential for bias.

After determining which studies to include, SNP used a standardized form to extract several objectively verifiable characteristics of the studies: 1) the number of participants and their composition by ethnicity, nativity, or country of origin (when available), lung cancer type, and lung cancer stage; 2) length of follow-up; 3) research design; 4) any covariates included in models; 5) comparison in OS and/or CSS; and 6) data source used. Once completed, the data extraction form was checked by MF. The Newcastle-Ottawa scale (NOS) (18), a quality assessment tool for use in nonrandomized studies included in systematic reviews, was used as the quality indicator. The NOS is among the most widely-used tools for assessing nonrandomized studies and has been previously endorsed for use in systematic reviews of nonrandomized (cohort and case-control) studies by the Cochrane Collaboration (19).

### Interpretation

We considered models to be supportive of the HHP if, after adjustment for key factors (age and stage at diagnosis), hazard ratios for Hispanic survival (compared with the NHW reference) were not greater than 1, and their respective upper and lower 95% confidence interval (CI) limits were below 1 (Hispanic advantage) or contained 1 (no statistical difference between Hispanic and NHW).

## Results

### Trial Flow

**Figure 1.**
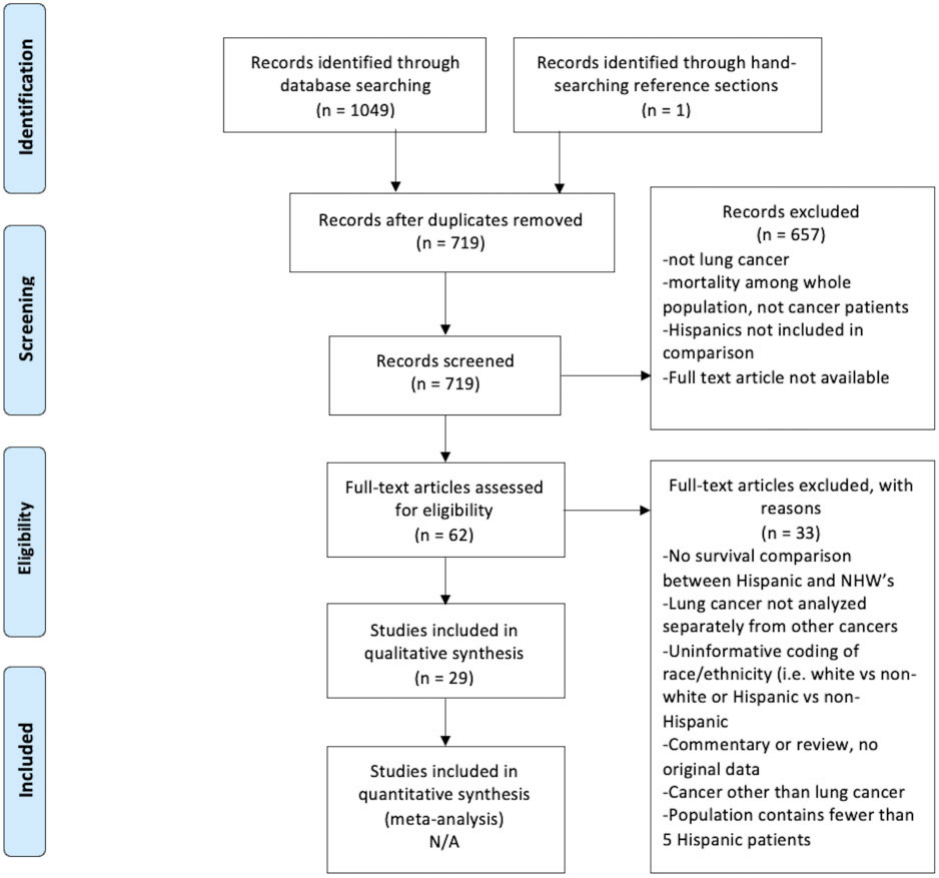
Preferred Reporting Items for Systematic Reviews and Meta-Analyses (PRISMA) flow diagram. NA = not applicable; NHW= non-Hispanic White

Electronic searches identified 1049 citations (see Figure 1). Hand-searching reference sections of included papers identified 1 additional citation. After duplicates were removed, 719 unique abstracts remained. All 719 abstracts were reviewed for inclusion. Based on titles and abstracts, 658 studies were excluded, 62 full-text articles were reviewed, and the authors of 13 conference abstracts were contacted to determine availability of published data based on these potentially eligible abstracts; 33 of the 62 full-text articles and 13 abstracts were excluded based on entry criteria. One of the 13 abstracts resulted in a publication dated after the initial search date, which was then included in the study. This search method yielded a final sample of 29 full-text articles.

### Study Characteristics

Data were reported from 8 unique data sources: various subsets of the SEER database (14 studies), the California Cancer Registry (8 studies), the Lung Cancer Mutation Consortium (1 study), the Cancer Care Outcomes & Surveillance Consortium (2 studies), the San Francisco Bay Area Lung Cancer Study (1 study), the Nevada Cancer Registry (1 study), the New Jersey State Cancer Registry (1 study), and the Department of Defense Automated Central Tumor Registry (1 study). Data collected from the final 29 studies spanned from 1973 to 2013, although publication dates ranged from 2002 to 2018. Although intervention studies were considered for inclusion in this review, no intervention studies met criteria because all had insufficient numbers to detect differences in survival by race and ethnicity (most studies enrolled fewer than 5 Hispanic participants). The majority (25) of included articles used a retrospective cohort study design, and 4 used a prospective cohort study design.

Of the 29 studies, 4 contained datasets that were entirely or mostly contained within larger datasets used by other studies (20‐23). These studies were nevertheless included as they reported on specific subpopulations or reported slightly different variable adjustment in their survival analyses.

### Characteristics of Participants

In 6 of the 29 studies, the exact number of Hispanic and NHW participants included in regression models was unclear, although estimates could be calculated from percentages in 2 studies (24‐29). In the remaining studies reporting numbers of both Hispanic and NHW participants (excluding the 4 completely overlapping studies noted above), there were 946 070 NHW participants and 78 327 Hispanic participants, with the number of Hispanics included in individual studies ranging from 28 to 18 206 and the number of NHWs ranging from 163 to 137 321. Although it is impossible to determine the exact number of unique participants involved because the majority of studies used the same or similar databases in overlapping years, the most conservative estimate (including only nonoverlapping studies) would indicate at least 186 386 unique cases for OS models (174 176 NHW and 12 210 Hispanic patients) and 208 063 unique cases for CSS models (191 511 NHW and 16 552 Hispanic patients). This estimate was reached by examining a combination of study data sources, years of data collection, and inclusion criteria. Study results from nonoverlapping studies only are presented in Supplementary Figure 1 (available online ).

Among the 29 included studies, 17 included only NSCLC cases, 1 included only SCLC, and the remainder (11) were inclusive of all histologic lung cancer types. Five studies included only those with early stage or local disease (stages I-II) at time of diagnosis, 4 included only those with advanced stages (III and/or IV), 3 included stages I-III, and the remainder (17) included participants with any stage of disease.

Across 8 studies reporting trends in histology by race and ethnicity, Hispanics were overrepresented among bronchioloalveolar carcinoma (2 studies) (26,30) and adenocarcinoma subtypes (5 studies) (22,24,31‐33), which are more common among nonsmokers and tend to be associated with better prognoses (30). Gomez and colleagues (34) found no difference in tumor characteristics by race and ethnicity, however. Compared with US-born Hispanics, foreign-born Hispanics were more likely to be diagnosed at younger ages, with more advanced disease, and were less likely to receive surgery, chemotherapy, or radiation (35).

Only 3 studies (30,34,35) collected self-reported nativity data (eg, US-born vs foreign-born Hispanic), and only 1 of these (30) classified Hispanics by native country. Of 30 papers, 6 (20.0%) reported participants’ smoking history (21,34,36‐39), and only 1 (39) reported on proportion of different oncogenic drivers in NHW and Hispanic populations. These limitations likely reflect the reliance on SEER data, which does not report these variables and made up nearly half (48.3%) of the included studies.

### Quality of the Included Studies

All but 4 studies used retrospective database cohort designs. Four studies used prospective cohort designs. The NOS, a risk of bias assessment tool for observational studies recommended by the Cochrane Collaboration, was used to assess study quality. The NOS assigns a maximum of 9 points for the smallest risk of bias in the following domains: selection of cohort (4 points), comparability of cohorts (2 points), and assessment of outcome (3 points). Regardless of study design, the studies were of high quality, with all scoring 7 or greater out of 9 on the NOS (a score ≥5 is considered high quality and low bias risk). Using the NOS, the studies scored on average 3.6 out of 4 stars for cohort selection, 1.9 of 2 stars for cohort comparability, and 2.3 of 3 stars for assessment of outcome. The most common reason for lost points was lacking a statement about the number of subjects lost to follow-up, followed by failing to control for age and stage at diagnosis. Only 1 study did not receive a star for representativeness because this study was conducted in a military population (38).

### Survival

**Figure 2.**
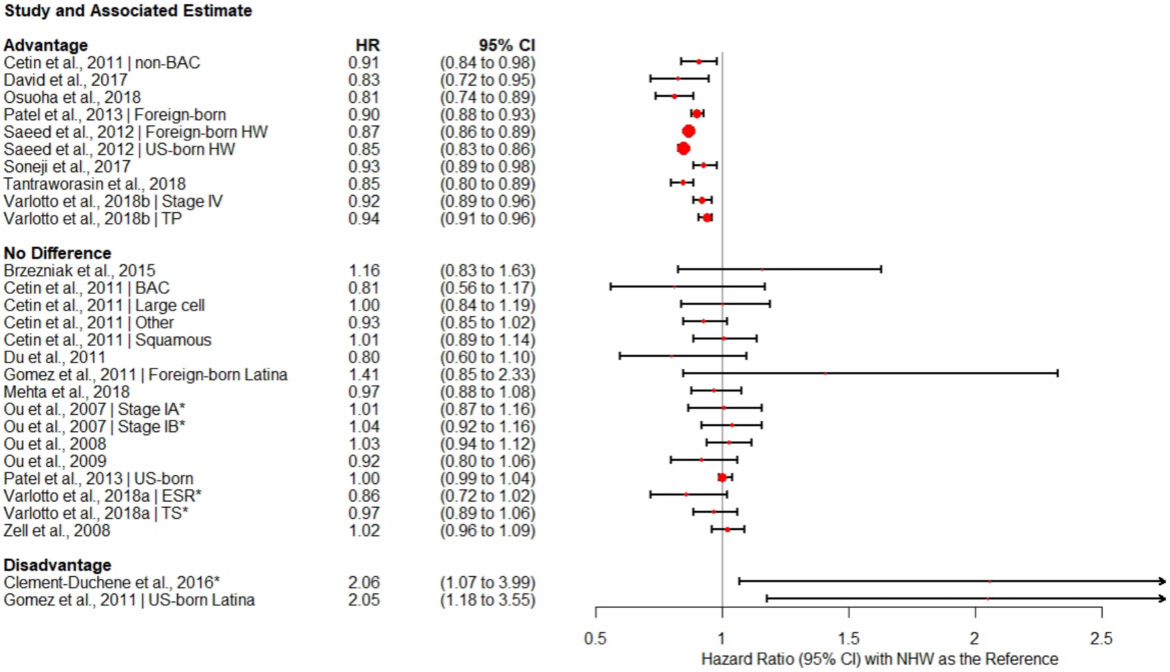
Forest plot of overall survival hazard ratios and 95% confidence intervals*.* Shorter confidence intervals (**error bars**) correspond to larger, more precise estimates. **Circles** correspond with effect size, with larger circles indicating a larger effect. **Asterisk** indicates the study population is entirely (or almost entirely) overlapping with another included study. CI = confidence interval; BAC = bronchioalveolar carcinoma; ESR = early stage resectable; HR = hazard ratio; HW = Hispanic White; NHW = non-Hispanic White; TP = total population.

**Figure 3.**
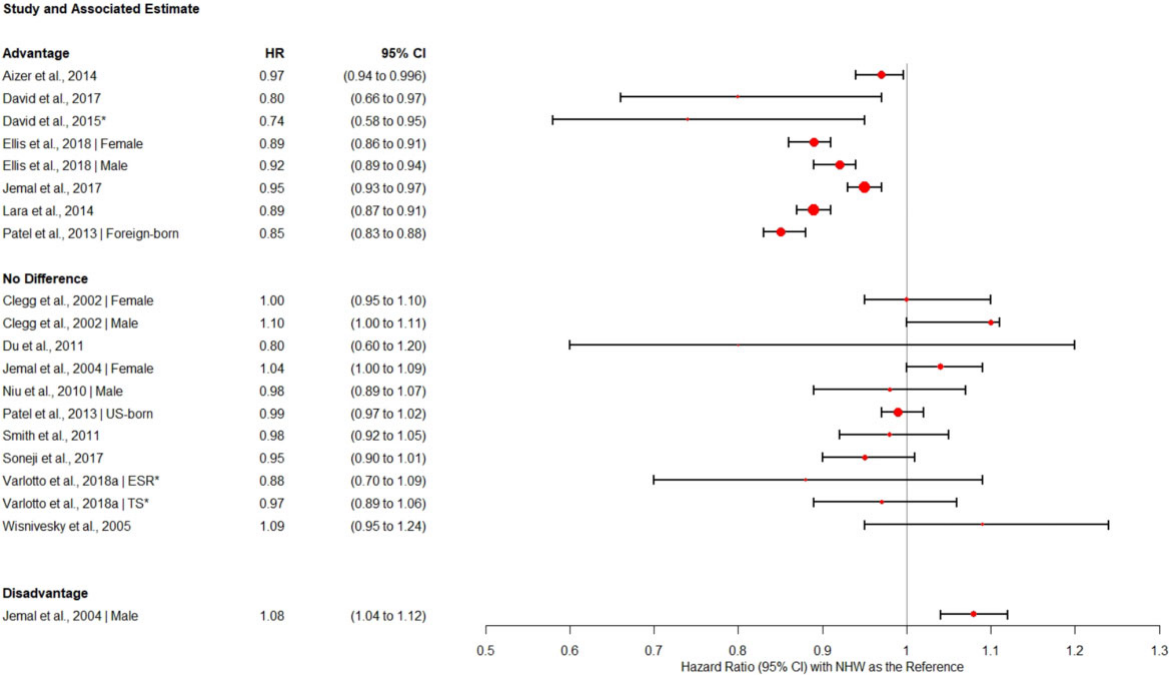
Forest plot of cancer-specific survival hazard ratios and 95% confidence intervals. Shorter confidence intervals (**error bars**) correspond to larger, more precise estimates. **Circles** correspond with effect size, with larger circles indicating a larger effect. **Asterisk** indicates the study population is entirely (or almost entirely) overlapping with another included study. CI = confidence interval; ESR = early stage resectable; HR = hazard ratio; NHW = non-Hispanic White; TS = total surgical population.

A total of 113 different models comparing OS and CSS for Hispanics vs NHWs were presented across the 29 studies. The models with the most covariates from each paper are presented in Figures 2 and 3, and the covariates included in these models are presented in Table 1. Hazard ratios and 95% confidence intervals were not included from 1 study (39) because of its relatively small sample size, minimal model adjustment, and wide 95% confidence intervals, which limited comparison with other studies. It is also important to note that many studies did not include *P* values along with hazard ratios and their 95% confidence intervals, so a model was considered supportive of a survival advantage if the confidence interval was below 1.0, supportive of no difference if the confidence interval included 1, and supportive of a disadvantage if the confidence interval range was above 1.0. Selecting fully adjusted models from each paper resulted in 28 models predicting OS and 21 predicting CSS. From the 29 included studies, 28 fully adjusted models evaluated ethnic differences in OS (20,22,26,29‐32,34‐38,41,42,45,46,48). From the 29 included studies, 21 evaluated ethnic differences in CSS (22‐25,27,28,33,35,40‐44,46,47). Note that some studies presented both estimates.

**Table 1.** Covariates included in final model for each study

Study	Covariates and hazard ratios (95% CI) of unadjusted or minimally adjusted models	Covariates in final model	Survival measure(s)	Sample considerations
Aizer et al., 2014 (40)	Not applicable, final adjusted model is only model reported	Age, race, income, education, urban vs rural residence, cancer stage, use of definitive therapy, sex	CSS	Nonmetastatic cancer only; SEER (1988-2007) data^a^; analytic sample: N = 165 505
Brzezniak et al., 2015 (38)	Not applicable, final adjusted model is only model reported	Treatment status (treated/not treated), age at diagnosis, race, sex, stage, histology, number of comorbidities, tobacco history, family history, marital status, region, alcohol history	OS	NSCLC only; Department of Defense Automated Central Tumor Registry (ACTUR); analytic sample: N = 4751
Cetin et al., 2011 (26)	Not applicable, final adjusted models are only models reported	Period of diagnosis, sex, age at diagnosis, ethnicity/race, tumor grade at diagnosis, receipt of cancer-directed surgery and radiation; separated by histology	OS	NSCLC only; First and only primary diagnosis of stage IV cancer; SEER (1988-2003) data; analytic sample: N = 51 749
Clegg et al., 2002 (25)	Not applicable, final adjusted model is only model reported	Age, tumor stage; separated by sex	CSS	SEER (1975-1997) data; analytic sample: N < 248 741^b^
Clément-Duchêne et al., 2016 (21)^c^	2.06 (1.07 to 3.00); adjusted for age, sex, race/ethnicity, comorbidity, insurance, income, education, health-care setting (non-integrated, integrated, VA), histology, stage, chemotherapy, radiotherapy	Age, sex, race/ethnicity, comorbidity, insurance, income, education, health-care setting (non-integrated, integrated, VA), histology, stage, chemotherapy, radiotherapy	OS	Never smokers only; Cancer Care Outcomes Research and Surveillance Study (CanCORS); analytic sample: N = 3410
David et al., 2015 (23)^c^	Not applicable, final adjusted model is only model reported	Age, sex, race, SES, tumor stage, types of treatment, hospital volumes, and ACS CoC accreditation	CSS	Stage 1 NSCLC only; California Cancer Registry (2004-2011) data; analytic sample: N = 7587
David et al., 2017 (41)	Not applicable, final adjusted models are only models reported	Lymph nodes examined, age, sex, race/ethnicity, SES, stage, tumor size, surgical treatment received	OS & CSS	Surgical resection patients only; California Cancer Registry (2004-2011) data; analytic sample: N = 16 393
Du et al., 2011 (42)	OS: 1.0 (0.8 to 1.2) and CSS: 1.0 (0.8 to 1.3); adjusted for age, sex, tumor stage, surgery, and radiotherapy	Age, sex, tumor stage, surgery, and radiotherapy, education, poverty, family income, health insurance	OS & CSS	National Longitudinal Mortality Study SEER (1973-2003) data; analytic sample: N < 13 234^d^
Ellis et al., 2018 (24)	Men: 1.08 (1.05 to 1.11); Women: 0.97 (0.95 to 1.00); adjusted for age	Baseline model included age, the covariates added one at a time based on degree of influence: stage at diagnosis, neighborhood SES, marital status, histology, hospital SES composition, surgery, tumor size, health insurance status, hospital insurance composition, NCI-designated cancer center, chemotherapy, year of diagnosis, neighborhood racial/ethnic composition, radiotherapy; separated by sex	CSS	California Cancer Center Registry (2000-2013) data; analytic sample: N = 181 060
Gomez et al., 2011 (34)	Not applicable, final adjusted models are only models reported	Age at diagnosis, race/ethnicity, marital status, histology, radiation, surgery, chemotherapy-by-time interaction; separate baseline hazards estimated by stage at diagnosis; separated by nativity status	OS	Women only; never-smokers only; Greater Bay Area Cancer Registry (1998-2003; 2005-2008) data; analytic sample: N = 472
Jemal et al., 2004 (27)	Not applicable, final adjusted model is only model reported	Age, tumor stage; separated by sex	CSS	SEER (1992-2000) data; analytic sample not reported
Jemal et al., 2017 (28)	Not applicable, final adjusted model is only model reported	Sex, age, summary stage	CSS	SEER (1975-1977; 2006 -2012) data; analytic sample not reported
Lara et al., 2014 (43)	Not applicable, final adjusted model is only model reported	Age (younger than 50 years or older than 50 years), sex, race/ethnicity, disease stage, treatment, year of diagnosis, SES, rural vs urban, histology	CSS	NSCLC only; California Cancer Center Registry (1998-2009) data; analytic sample: N = 114 451
Mehta et al., 2018 (37)	1.08 (0.99 to 1.19); unadjusted	Race/ethnicity, income, education, insurance, sex, age at diagnosis, marital status, integrated health system (yes/no), stage, comorbidities (0, 1, 2, 3, 4+), smoking status, radiation (yes/no), chemo (yes/no), surgery (yes/no)	OS	NSCLC only; Cancer Care Outcomes Research Surveillance (2003-2005) data; analytic sample: N = 3250
Niu et al., 2010 (44)	Men: 1.04 (0.95 to 1.13), Women: 0.92 (0.82 to 1.04); stratified by age and stage at diagnosis	Race/ethnicity and area poverty level, stratified by age and stage at diagnosis; separated by sex	CSS	New Jersey State Cancer Registry (1986-1999) data; analytic sample: N = 37 765
Osuoha et al., 2018 (32)	Not applicable, final adjusted model is only model reported	Sex, age, race/ethnicity, Nevada region, marital status, stage at diagnosis, histology	OS	Nevada Central Cancer Registry (2003-2010) data; analytic sample: N = 12 964
Ou et al., 2007 (20)	Not applicable, final adjusted models are only models reported	Period of diagnosis (1989-1993, 1994-1998, 1999-2003), ethnic origin, location of tumor, histology, tumor size, histologic grade, age at diagnosis, SES quintile, sex, surgery, radiation, chemotherapy; separate models for stage IA and stage IB	OS	NSCLC only; stage I only; California Cancer Center Registry (1989-2003) data; analytic sample: N = 19 702
Ou et al., 2008 (45)^c^	1.105 (1.01 to 1.21); adjusted for ethnic origin, radiation, chemotherapy, age at diagnosis, sex, histology, histologic grade, and tumor lobar location	Ethnic origin, radiation, chemo, surgery, marital status, SES quintile, age at diagnosis, sex, histology, histologic grade, and tumor lobar location	OS	NSCLC only; stage I only; California Cancer Center Registry (1989-2003) data; analytic sample: N = 19 702
Ou et al., 2009 (36)	Not applicable, final adjusted model is only model reported	Smoking history (yes/no), ethnicity, sex, age, SES, marital status (married, unmarried), chemotherapy (no/yes), radiation (no/yes), surgery (no/yes)	OS	SCLC only; Cancer Surveillance Programs of Orange, San Diego and Imperial counties in California (1991-2005) data; analytic sample: N = 3428
Patel et al., 2013 (35)	US-born: 1.31 (1.00 to 1.06), Foreign-born: 0.92 (0.90 to 0.95); adjusted for age, sex, stage, histology, year of diagnosis, marital status	Age, sex, stage, histology, year of diagnosis, marital status, surgery, radiation, and chemotherapy, neighborhood SES/ethnic enclave; separated by nativity status	OS & CSS	NSCLC only; California Cancer Center Registry (1988-2007) data; analytic sample: N = 151 601
Saeed et al., 2012 (30)	Not applicable, final adjusted models are only models reported	Age, sex, histology, stage, race/ethnicity	OS	NSCLC only; SEER (1988-2007) data; analytic sample: N = 172 398
Smith et al., 2011 (33)	1.05 (0.98 to 1.12); adjusted for age, sex, marital status, histology	Age, sex, marital status, stage at diagnosis, surgery, radiation therapy use	CSS	NSCLC only; stages I-IIIA only; SEER (1988-2006) data; analytic sample: N = 69 138
Soneji et al., 2017 (46)	Not applicable, final adjusted model is only model reported	Race/ethnicity, sex, age at diagnosis, histologic type, stage at diagnosis, type of surgical resection, radiation sequence (before surgery, after surgery, before and after, sequence unknown)	OS & CSS	Early stages (IA, IB, IIA, IIB) only; SEER (2004-2013) data; analytic sample: N = 105 121
Steuer et al., 2016 (39)^a^	*EGFR*: 1.00 (0.36 to 2.76); *KRAS*: 2.08 (0.51 to 8.43); *ALK*: 1.14 (0.15 to 8.42); *MET*: 1.53 (0.18 to 12.82); *PIK3CA*: 1.03 (0.13 to 8.39); *BRAF*: 1.01 (0.11 to 8.86); unadjusted	Race/ethnicity	OS	Lung Cancer Mutation Consortium data; analytic sample: N = 1007
Tantraworasin et al., 2018 (29)	Not applicable, final adjusted model is only model reported	Ethnicity, insurance type, adjusted by patient demographic, pathologic result, stage of disease, cancer-specific treatment	OS	Ages 18-64 years only; SEER (2007-2013) data; analytic sample not reported for analyses with Hispanic patients
Varlotto et al., 2018 (22)^c^	Not applicable, final adjusted models are only models reported	Race, reporting registry, sex, age, income, marital status, tumor size, tumor stage, insurance, lateral location, histology, grade, surgical procedure, radiation post-operative, number of nodes examined, node density, year of diagnosis; early stage resectable and total surgical population ran separately	OS & CSS	Surgically resected NSCLC only; SEER-18 (2007-2012) data; analytic sample: N = 35 689
Varlotto et al., 2018 (31)	Not applicable, final adjusted models are only models reported	Age, race, SEER registry, income, insurance, marital status, tumor stage, lateral location, histology, grade, definitive surgical procedure, radiation, year of diagnosis; total population and stage IV only ran separately	OS	SEER-18 (2007-2012); analytic sample: N = 70 968
Wisnivesky et al., 2005 (47)	1.23 (1.08 to 1.41); adjusted for age, sex, marital status, and mean income in the geographic area	Age, sex, marital status, and mean income in the geographic area, surgical treatment received, and stage at diagnosis (IA vs IB)	CSS	Stage 1 NSCLC only; SEER (1991-2000) data; analytic sample: N = 16 036
Zell et al., 2008 (48)	Not applicable, final adjusted model is only model reported	Stage, age at diagnosis, sex, ethnicity/race, surgical treatment status, radiotherapy	OS	NSCLC only; stages IIIB and IV only; SEER (1999-2003) data; analytic sample: N = 27 435

a Paper not included in forest plots. ACS CoC = American College of Surgeons Commission on Cancer; CI = confidence interval; CSS = cause-specific survival; NCI = National Cancer Institute; NSCLC = non-small cell lung cancer; OS = overall survival; SCLC = small cell lung cancer; SEER = Surveillance, Epidemiology, and End Results Program; SES = socioeconomic status; VA = Veterans Affairs.

b Analytic sample for survival analyses not available.

c Study population is entirely (or almost entirely) overlapping with another included study.

d Sample number for lung cancer patients not available.

### Overall Survival

In 28 models, 26 (92.9%) documented either no difference or a Hispanic advantage in OS. This includes 10 (35.7%) models that demonstrated an OS advantage for Hispanic patients compared with NHWs [6.0%-19.0% lower risk of death; see Figure 2 (26,29‐32,35,41,46)] and 16 (57.1%) models (20,26,34‐38,42,45) that showed no difference between Hispanics and NHWs. Only 2 models showed a Hispanic disadvantage in OS (at any given time *t,* Hispanics experienced mortality at approximately twice the rate of NHWs) (21,34). Notably, both of the disadvantage models were from studies of exclusively never-smokers. When examining SEER studies alone, 7 of 19 (36.8%) models documented a Hispanic OS advantage, and the remaining 12 (63.2%) documented no overall survival difference between Hispanics and NHWs.

Several trends emerged within the data. As expected, studies with the largest patient samples yielded the narrowest confidence intervals. These studies were also most likely to document Hispanic survival advantages and to be derived from SEER or the California Cancer Registry (30,31,35,46). For example, including only studies with clear sample sizes, the mean sample size in the Hispanic advantage models was approximately 2.6 times larger than the mean samples from the no-difference models: 77 887 (range = 12 964-17 238) vs 31 778 (range = 472-151 601). These proportional differences in sample size are important in considering strength of study findings. Of note, the 2 studies reporting the greatest magnitude of survival difference between Hispanics and NHWs (Hispanic disadvantage; HR > 2.00) (21,34) were also among the smallest samples and consisted of never-smokers only, whereas the largest studies included more representative samples and reported a smaller magnitude of difference in the direction of a Hispanic survival advantage (HR = 0.85-0.94).

A small number of models reported nativity effects on overall survival. Two models from large studies with samples in excess of 150 000 evaluated survival differences between foreign-born Hispanics and NHWs, with both showing a robust Hispanic advantage (10.0%-13.0% more likely to be alive at study follow-up; HR = 0.87 and 0.90, respectively) (30,35). In contrast, Gomez et al. (34) showed no difference among foreign-born Latinas and NHW women in a relatively small sample of 472 patients (HR = 1.41, 95% CI = 0.85 to 2.33). Again, none of the 3 models documented a disadvantage for foreign-born Hispanics.

Three models tested OS differences between US-born Hispanics and NHWs. These studies documented mixed results, with the largest (30) showing a Hispanic advantage of approximately 15.0% (HR = 0.85, CI = 0.83 to 0.86) and a second analysis from the Patel et al. (35) study showing no difference (HR = 1.00, 95% CI = 0.99 to 1.04). The third model, as reported by Gomez et al. (34), notes a clear disadvantage (HR = 2.05, 95% CI = 1.18 to 3.55).

### Cause-Specific Survival

In 21 models, 20 (95.2%) documented either no difference or a Hispanic advantage in cancer-specific mortality. Nine (14.9%) of the 21 models demonstrated a CSS advantage for Hispanic patients compared with NHWs (3.0%-60.0% lower risk of death given respective follow-ups; see Figure 3) (23,24,28,35,40,41,43,44). Of the 21 models, 11 (52.4%) showed no difference between Hispanics and NHWs (22,25,27,33,35,42,44,46,47). Only 1 model found a Hispanic disadvantage in cancer-specific survival (8.0% higher risk of death) but only among men (27). When examining SEER studies alone, 2 (16.7%) of 12 models documented a Hispanic CSS advantage, 9 (75.0%) documented no cause-specific survival difference, and 1 (8.3%) documented a survival disadvantage for Hispanics with lung cancer compared with NHWs.

Similar to the OS findings, studies and models with the largest patient samples generally yielded the narrowest confidence intervals and demonstrated the strongest Hispanic CSS advantage compared with NHWs (24,28,35,40,43). Including only studies with clear sample sizes, the mean sample size in the models reporting an advantage was approximately 1.4 times larger than the mean of the models reporting no difference (96 337 [range = 7587-181 060] vs 69 225 [range = 16 036-151 601]). Again, studies with the largest sample sizes generally demonstrated a smaller magnitude of survival difference between Hispanics and NHWs in the direction of an advantage (HR = 0.89-0.97) (24,35,40,43).

Only 2 models tested nativity differences in cancer-specific survival compared with NHWs. Both models stem from the study by Patel et al. (35) and mirror the trends observed for OS. Once again, US-born Hispanics were found to have similar cancer-specific survival, and foreign-born Hispanics were found to have lower cancer-specific mortality compared with NHWs (35).

### Model-Level Findings: Assessment of Covariates and Potential Mechanisms

Although we present only the most complex models from each study in Figures 2 and 3, 113 models in total were examined across the 29 studies. The majority of studies included adjusted models for age, sex, and stage at diagnosis, and several compared the influence of different covariates on racial and ethnic differences in survival. Of the models tested, 18 (15.9%) had minimal adjustment (unadjusted or adjusted only for age, sex, and stage), 12 (10.6%) had moderate adjustment (adjusted for demographic characteristics and clinical characteristics such as histology and treatment received), and the remaining 83 (73.5%) had extensive adjustment (controlling for SES, marital status, neighborhood characteristics, etc.). Although not all studies published unadjusted or minimally adjusted hazard ratios, when reported, models with minimal adjustment were less likely to show a Hispanic survival advantage (see Table 1). Eleven percent of models with minimal adjustment, 25.0% of models with moderate adjustment, and 50.0% of models with extensive adjustment were supportive of a Hispanic survival advantage. Broadly, only 9 (8.0%) of the 113 models tested showed a Hispanic survival disadvantage, 47 (41.6%) showed a survival advantage, and the remainder (57; 50.4%) showed no difference.

Generally, controlling for the effect of SES-related variables increased the effect size of hazard ratios depicting a Hispanic survival advantage, reduced any disparities for Hispanics to zero, or even reversed a disadvantage to an advantage (21,24,33,35,36,42,44,47). These findings underscore the importance of SES-related variables in understanding ethnic outcome differences in the context of lung cancer.

A handful of studies attempted to control for treatment disparities with mixed results. Some studies included in this review suggest that adding treatment variables into adjusted models eliminates disparities (35,47), others found that removing treatment variables eliminates disparities (21), and still others found that adding these variables makes no difference (34,38). These differences likely reflect heterogeneity in treatment variables within and across studies as well as the overall lack of associated critical information reported such as timing and quality of treatment.

Although stage at diagnosis is an important covariate to consider because Hispanics tend to be diagnosed at later stages of disease than NHWs, patterns appeared fairly consistent across all stages (see Supplementary Figures 2 and 3, available online, for OS and CSS by stage). One-quarter of models examining cohorts diagnosed with lung cancer at advanced stages (IIIB-IV) demonstrated a Hispanic survival advantage, and the remainder (75.0%) showed no difference in survival between NHW and Hispanics, which was identical to the ratio of advantage to no difference models found among early stage (I-IIB) patients. The majority of models including patients of all stages demonstrated either a Hispanic survival advantage (46.7%) or no difference (40.0%). Varlotto and colleagues (31) found a Hispanic survival advantage compared with NHWs when analyzing both the total population and stage IV patients alone. When comparing hazard ratios between different target populations examined using the same datasets (eg, SEER 9, SEER 13, SEER 18, overlapping years of the California Cancer Registry), results were generally consistent across lung cancer types and stages within data sources. In their examination of stage IV patients using SEER 9, Cetin et al. (26) and Clegg et al. (25) found no survival difference between NHWs and Hispanics for those with NSCLC or with any type of lung cancer. Among SEER 13 studies, Zell et al. (48) and Du et al. (42) also found consistent results between those diagnosed at advanced stages (IIIB-IV) and those diagnosed at any stage. Among California Cancer Registry studies, Ou and colleagues found similar results for SCLC and NSCLC (no survival difference) during overlapping years (approximately 1989-2005) (20,36,45). Comparing hazard ratios for 4 different subgroups of NSCLC patients examined across 2 studies using the SEER 18 dataset (stage IV only, total population, early stage resectable, and total surgical population) (22,31), it appears that the paradox may be more pronounced for patients with more advanced or inoperable disease, although the fact that hazard ratios for the surgical populations were adjusted using additional covariates (lateral location, number of nodes examined, node density) limits the strength of this conclusion.

Sex emerged as another potentially important covariate, with the Hispanic survival advantage appearing more pronounced in women than men. Ellis and colleagues (24) found that Hispanic men and women had a survival advantage compared with NHWs, but the magnitude of this effect was stronger for women, Niu and colleagues (44) found a Hispanic survival advantage for women but no survival difference for men, and Jemal and colleagues (27) found no survival difference for women but a disadvantage relative to NHWs for Hispanic men.

## Discussion

The 29 studies published over the last 2 decades overwhelmingly illustrate that Hispanics exhibit similar or better survival in the context of lung cancer relative to NHWs despite a substantially worse risk factor profile. These results strongly support the HHP in the context of lung cancer survival. Specifically, among fully adjusted models, 26 of 28 models (92.9%) evaluating overall survival and 20 of 21 (95.2%) evaluating cancer-specific mortality documented either no difference or a Hispanic survival advantage. Larger studies and those models comparing NHWs with foreign-born Hispanics were more likely to show a Hispanic survival advantage. Together, these findings extend the known health advantages associated with Hispanic ethnicity and support further investigation into resilience mechanisms both for lung cancer specifically and health outcomes broadly.

Several moderators (eg, variables that impact the strength and direction of a statistical relationship) emerged as critical to understanding the trends in differential risk. Generally, controlling for the effect of SES-related variables increased the effect size of hazard ratios depicting a Hispanic survival advantage, reduced any disparities for Hispanics to zero, or even reversed a disadvantage to an advantage. Despite limited data, nativity status also emerged as a reliable determinant of outcomes with foreign-born Hispanics experiencing some of the strongest advantages. These findings are particularly paradoxical given that foreign-born Hispanics tend to be among the lowest in SES and have relatively greater disparities in health-care resources compared with their US-born counterparts and to NHWs, generally (4). However, both the independent and combined effects of SES and nativity on survival outcomes are consistent with the broader findings on comparative Hispanic health.

Importantly, the observed findings were not contingent on stage of diagnosis. The majority of reviewed studies did not identify stage as an important moderator of findings, and comparable findings were noted in studies that focused on either early (I-IIA) or late (IIIB-IV) stage diagnostic categories. Hispanic lung cancer patients are more likely to be diagnosed at later stages. Yet, even within these later stages, their odds of survival remain comparable to advantageous.

This study also points to potential sex differences in the magnitude of the HHP, as some analyses separated by sex found that Hispanic women show a greater survival advantage relative to NHWs than their male counterparts. Future research should investigate whether the effect of Hispanic ethnicity on lung cancer survival is moderated by sex. Although a previous meta-analysis did not find a moderating effect of sex on the Hispanic mortality paradox (14) and challenged the generalizability of the relationship between SES and disease risk, the present finding that the HHP may be stronger among women with lung cancer also aligns with previous findings that the SES gradient appears to be stronger for men than for women across many health outcomes (49).

Despite our ability to make these specific observations, critical gaps in the literature must be noted. Most of the literature is reliant on a few key public health databases, which makes disaggregating for purposes of a meta-analysis problematic. These databases generally lack potentially important moderators, including standard markers of SES, stage of diagnosis, and Hispanic ancestral background. Hispanics are not a homogenous group, and the lack of associated demographic data undermines the specificity of knowledge and targeted intervention efforts.

The robust nature of the broader paradoxical outcomes supports a shift from questioning their validity to investigating potential mechanisms of action. A lower rate of smoking among Hispanics is an important factor to understand reduced lung cancer incidence, and this variance may also help explain mortality differences once diagnosed with lung cancer (50). This pathway is potentially supported by 2 studies of exclusively nonsmokers that showed a Hispanic mortality disadvantage. It is important to note that there are limited data regarding ethnic differences in smoking rates among lung cancer patients (51). However, the 3 studies included here report measures of smoking continue to support the paradox after controlling for that behavior. We can reasonably infer from this evidence that whatever differences in smoking exist, when accounted for, they do not fully explain the HHP in the context of lung cancer.

Moreover, differential smoking rates do not explain the broader HHP beyond lung cancer, which is evident in multiple studies controlling for smoking status and history. For example, a recent study of more than 161 000 postmenopausal women in the United States found a robust Hispanic mortality advantage even after controlling for smoking status (52). Recent research also shows that although elimination of key health-risk behaviors (eg, smoking, obesity) would reduce health disparities, the impact would be greater on NHB–NHW differences than on Hispanic–NHW differences (53,54). Thus, the aggregate evidence to date suggests that although smoking may impact the degree of Hispanic advantage, it does not account for the broader HHP or the specific trends in the context of lung cancer.

Lung cancer histology is also important to consider; Saeed and colleagues (30 ) noted higher rates of bronchioloalveolar carcinoma subtype among Hispanics, suggesting that Hispanics may be more likely to be diagnosed with lung cancer subtypes associated with never-smoker status and better prognoses. However, our analysis revealed multiple studies that controlled for histology and continued to report a mortality advantage for Hispanics. Only 1 relatively small study included in this review explored the prevalence of specific oncogenic drivers among Hispanics and NHWs with lung cancer (39). With the increasing prevalence of direct treatment strategies targeting specific mutations (eg, *EGFR)*, the presence of these oncogenic drivers may play a greater role in survival differences, because they may not be evenly distributed by race and ethnicity. Some research suggests that Hispanics have a higher prevalence of oncogenic drivers than NHWs, but these results are inconsistent and complicated by the fact that Hispanics comprise a racially and genetically diverse group, and rates of certain mutations like *EGFR* vary based on country of origin (55). These inconsistencies, coupled with the fact that Hispanics face substantial barriers to care (lower rates of treatment, underrepresentation in clinical trials), suggest that differential somatic mutations alone are insufficient to explain racial and ethnic disparities in lung cancer outcomes (55,56). Other proposed mechanisms, including ethnic differences in diet (eg, bean consumption) (57) and cellular aging (eg, epigenetic methylation) (58), have less empirical and conceptual support and are unlikely to meaningfully drive lung cancer mortality differences.

Within the broader Hispanic health literature, the dominant hypothesis to explain Hispanic health advantages concerns the role of cultural factors facilitating social integration; these mechanisms must be seriously considered in the context of lung cancer as well (11). Social integration is among the most robust psychosocial determinants of a range of objective health outcomes, with a strong subliterature demonstrating effects on objective outcomes including mortality (59). Indeed, meta-analytic findings suggest that greater social integration rivals reduced smoking in terms of mortality impact (59). Social integration may be a particularly impactful resilience factor for Hispanics, given cultural values of collectivism and family (referred to as familismo), and interpersonal harmony (referred to as simpatía). Moreover, such values may be stronger in less acculturated Hispanic patients contributing to the consistently observed nativity benefits. In the context of lung cancer, cultural moderation of social pathways may engender a more sustained postdiagnosis integration benefit leading to clinical advantages for Hispanic lung cancer patients. Direct examination of this hypothesis is a critical next step in understanding mortality benefits and in pursuing culturally informed interventions focused on resiliency and social integration.

In summary, the current review strongly supports Hispanic lung cancer outcomes as generally consistent with the HHP. These findings further challenge generalized notions of minority health and of standard predictive models and should motivate efforts to elucidate potential resilience mechanisms.

## Funding

This research was supported in part by a grant from the National Cancer Institute (R01CA262719-01) awarded to Dr Ruiz and Dr Hamann.

## Notes

**Role of the funder:** The funder had no role in the study design, collection, analysis, or interpretation of data, or preparation and submission of the manuscript.

**Authordisclosures:** The authors have no disclosures to report.

**Author contributions:** SNP: conceptualization, methodology, validation, data curation, writing-original draft, writing- review & editing, project administration. MF: conceptualization, methodology, validation, data curation, visualization, writing- review & editing. HAH: conceptualization, methodology, writing- review & editing. JMR: conceptualization, methodology, validation, writing- review & editing, project administration, supervision, resources.

**Priorpresentations:** This work has been presented in part at the Society of Behavioral Medicine (April 11-14, 2018) and American Psychosomatic Society (March 9-12, 2019) Annual Meetings.

## Data Availability

All data are incorporated into the article and its online Supplementary Material.

## Supplementary Material

pkab062_Supplementary_Data
