# Supplementary material for: Ethnic Differences in Survival Among Lung Cancer Patients: A Systematic Review
Source: JNCI Cancer Spectr. 2021 Jul 7;5(5):pkab062. doi: 10.1093/jncics/pkab062 (PMC8410140; doi:10.1093/jncics/pkab062)
Supplement: pkab062_Supplementary_Data [file pkab062_supplementary_data.pdf]

# SUPPLEMENTARY MATERIALS

## Supplementary Methods

### Search Strategy Example- PubMed/MEDLINE

01. Hispanic
02. Latino
03. Mexic*
04. Cuban*
05. Puerto Ric*
06. “Lung cancer”
07. “thoracic neoplasm”
08. “non-small cell lung cancer”
09. “non small cell lung cancer”
10. “small cell lung cancer”
11. “thoracic neoplasms”
12. 1 OR 2 OR 3 OR 4 OR 5
13. 6 OR 7 OR 8 OR 9 OR 10 OR 11

Limits: only items with abstracts, humans, English, publication date from 2000-2018

**Supplementary Figure 1: Forest Plot of Overall and Cancer-Specific Survival Hazard Ratios and 95% CIs from Selected Non-Overlapping Studies.**

**Study and Associated Estimate**

| Study | HR | 95% CI |
|---|---|---|
| **Overall Survival: Advantage** | **HR** | **95% CI** |
| Cetin et al., 2011 \| non-BAC[1] | 0.91 | (0.84, 0.98) |
| Osuoha et al., 2018[2] | 0.81 | (0.74, 0.89) |
| Varlotto et al., 2018b \| Stage IV[3] | 0.92 | (0.89, 0.96) |
| Varlotto et al., 2018b \| TP[3] | 0.94 | (0.91, 0.96) |
| **Overall Survival: No Difference** | | |
| Cetin et al., 2011 \| BAC[1] | 0.81 | (0.56, 1.17) |
| Cetin et al., 2011 \| Large Cell[1] | 1.00 | (0.84, 1.19) |
| Cetin et al., 2011 \| Other[1] | 0.93 | (0.85, 1.02) |
| Cetin et al., 2011 \| Squamous[1] | 1.01 | (0.89, 1.14) |
| Ou et al., 2007 \| Stage IA*[4] | 1.01 | (0.87, 1.16) |
| Ou et al., 2007 \| Stage IB*[4] | 1.04 | (0.92, 1.16) |
| Ou et al., 2009[5] | 0.92 | (0.80, 1.06) |
| **Cancer Specific Survival: Advantage** | **HR** | **95% CI** |
| Patel et al., 2013 \| Foreign-born[6] | 0.85 | (0.83, 0.88) |
| Niu et al., 2010 \| Female[7] | 0.88 | (0.78, 0.99) |
| **Cancer Specific Survival: No Difference** | | |
| Patel et al., 2013 \| US-born[6] | 0.99 | (0.97, 1.02) |
| Niu et al., 2010 \| Male[7] | 0.98 | (0.89, 1.07) |

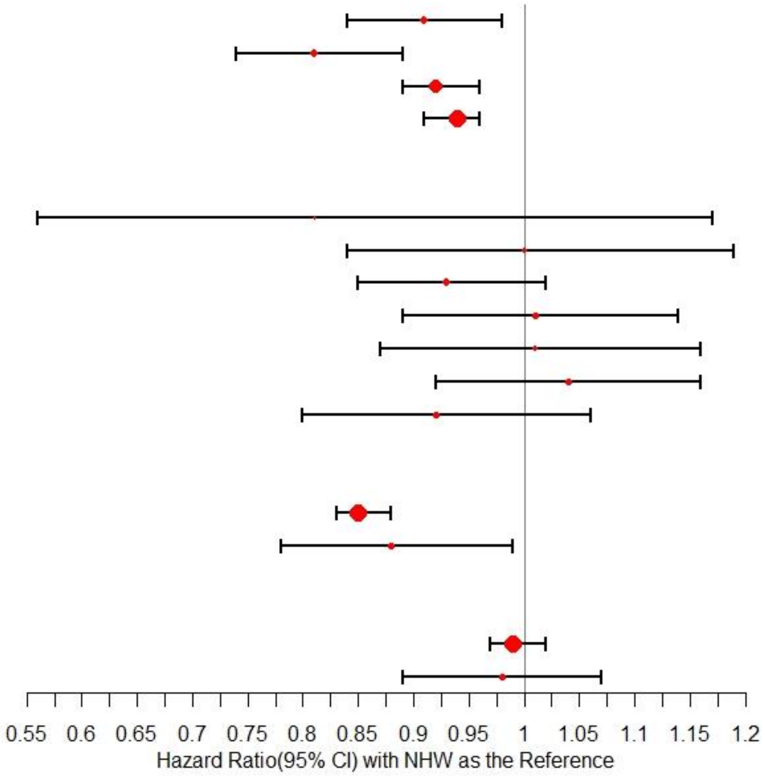


Non-overlapping studies were determined by examining study data sources, years of data collection, and inclusion criteria. In the case were multiple studies used the same data source from partially or entirely overlapping years, we selected a combination of studies from that data source that allowed us to explore the greatest number of unique cases. Shorter confidence intervals (error bars) correspond to larger, more precise estimates. Circles correspond with effect size, with larger circles indicating a larger effect. **Asterisk** indicates the study population is entirely (or almost entirely) overlapping with another included study.

Non-BAC=non-bronchioalveolar carcinoma; TP= total population; BAC= bronchioalveolar carcinoma; US= United States

**Supplementary Figure 2: Forest Plot of Overall Survival Hazard Ratios and 95% CIs by Cancer Stage.**

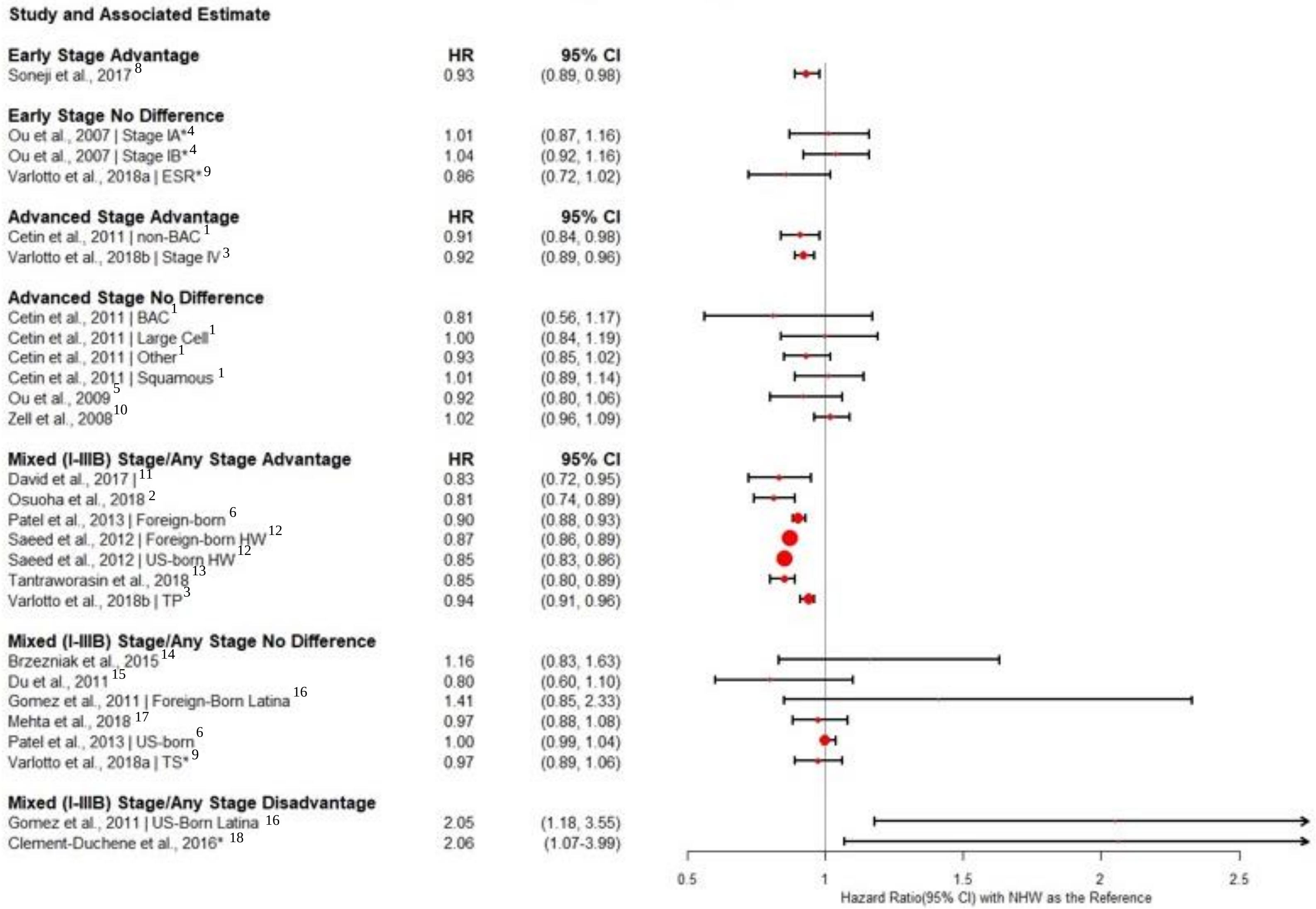


Shorter confidence intervals (error bars) correspond to larger, more precise estimates. Circles correspond with effect size, with larger circles indicating a larger effect. **Asterisk** indicates the study population is entirely (or almost entirely) overlapping with another included study. Early stage =Stage I-IIA; Advanced Stage =Stage IIIB-IV.

ESR= Early stage resectable; non-BAC=non-bronchioalveolar carcinoma; BAC=bronchioalveolar carcinoma; US= United States; HW= Hispanic white; TP= total population; TP= total population; TS= total surgical population

**Supplementary Figure 3: Forest Plot of Cancer-Specific Survival Hazard Ratios and 95% CIs by Cancer Stage.**

| Study and Associated Estimate | | |
|---|---|---|
| **Early Stage (I-IIIA) Advantage** | **HR** | **95% CI** |
| David et al., 2015* [19] | 0.74 | (0.58, 0.95) |
| **Early Stage (I-IIIA) No Difference** | | |
| Smith et al., 2011 [20] | 0.98 | (0.92, 1.05) |
| Soneji et al., 2017 [8] | 0.95 | (0.90, 1.01) |
| Varlotto et al., 2018a \| ESR* [9] | 0.88 | (0.70, 1.09) |
| Wisnivesky et al., 2005 [21] | 1.09 | (0.95, 1.24) |
| **Advanced (IIIB-IV)** | | |
| None | | |
| **Mixed (I-IIIB)/Any Stage Advantage** | **HR** | **95% CI** |
| Aizer et al., 2014 [22] | 0.97 | (0.94, 0.996) |
| David et al., 2017 [11] | 0.80 | (0.66, 0.97) |
| Ellis et al. , 2018 \| Female [23] | 0.89 | (0.86, 0.91) |
| Ellis et al. , 2018 \| Male [23] | 0.92 | (0.89, 0.94) |
| Jemal et al., 2017 [24] | 0.95 | (0.93, 0.97) |
| Lara et al., 2014 [25] | 0.89 | (0.87, 0.91) |
| Niu et al., 2010 \| Female [7] | 0.88 | (0.78, 0.99) |
| Patel et al., 2013 \| Foreign-born [6] | 0.85 | (0.83, 0.88) |
| **Mixed (I-IIIB)/Any Stage No Difference** | | |
| Niu et al., 2010 \| Male [7] | 0.98 | (0.89, 1.07) |
| Patel et al., 2013 \| US-born [6] | 0.99 | (0.97, 1.02) |
| Clegg et al., 2002 \| Female [26] | 1.00 | (0.95, 1.10) |
| Clegg et al., 2002 \| Male [26] | 1.10 | (1.00, 1.11) |
| Du et al., 2011 [11] | 0.80 | (0.60, 1.20) |
| Jemal et al., 2004 \| Female [27] | 1.04 | (1.00, 1.09) |
| Varlotto et al., 2018a \| TS* [9] | 0.97 | (0.89, 1.06) |
| **Mixed (I-IIIB)/Any Stage Disadvantage** | | |
| Jemal et al., 2004 \| Male [27] | 1.08 | (1.04, 1.12) |

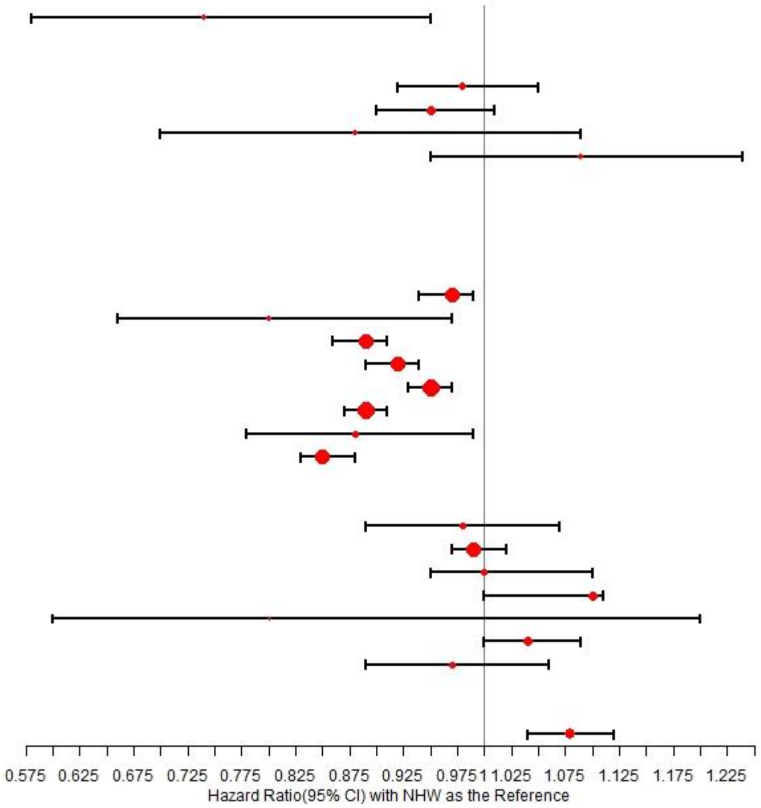


Shorter confidence intervals (error bars) correspond to larger, more precise estimates. Circles correspond with effect size, with larger circles indicating a larger effect. **Asterisk** indicates the study population is entirely (or almost entirely) overlapping with another included study. Early stage =Stage I-IIA; Advanced Stage =Stage IIIB-IV; Mixed/Any Stage= Stage I-IIIB or any stage.

ESR= Early stage resectable; US= United States; TS= total surgical population

## References


1. Cetin K, Ettinger DS, Hei Y-J, O'Malley C. Survival by histologic subtype in stage IV nonsmall cell lung cancer based on data from the Surveillance, Epidemiology and End Results Program. *Clin Epidemiol*. 2011;3:139. doi:10.2147/CLEP.S17191
2. Osuoha CA, Callahan KE, Ponce CP, Pinheiro PS. Disparities in lung cancer survival and receipt of surgical treatment. *Lung Cancer*. 2018;122:54-59. doi:10.1016/j.lungcan.2018.05.022
3. Varlotto JM, Voland R, McKie K, et al. Population-based differences in the outcome and presentation of lung cancer patients based upon racial, histologic, and economic factors in all lung patients and those with metastatic disease. *Cancer Med*. 2018;7(4):1211-1220. doi:10.1002/cam4.1430
4. Ou S-HI, Zell JA, Ziogas A, Anton-Culver H. Prognostic factors for survival of stage I nonsmall cell lung cancer patients. *Cancer*. 2007;110(7):1532-1541. doi:10.1002/cncr.22938
5. Ou S-HI, Ziogas A, Zell JA. Prognostic Factors for Survival in Extensive Stage Small Cell Lung Cancer (ED-SCLC): The Importance of Smoking History, Socioeconomic and Marital Statuses, and Ethnicity. *J Thorac Oncol*. 2009;4(1):37-43. doi:10.1097/JTO.0b013e31819140fb
6. Patel MI, Schupp CW, Gomez SL, Chang ET, Wakelee HA. How Do Social Factors Explain Outcomes in Non–Small-Cell Lung Cancer Among Hispanics in California? Explaining the Hispanic Paradox. *J Clin Oncol*. 2013;31(28):3572-3578. doi:10.1200/JCO.2012.48.6217
7. Xiaoling Niu X, Karen S. Pawlish KS, Lisa M. Roche LM. Cancer Survival Disparities by

Race/Ethnicity and Socioeconomic Status in New Jersey. *J Health Care Poor Underserved*. 2010;21(1):144-160. doi:10.1353/hpu.0.0263

8. Soneji S, Tanner NT, Silvestri GA, Lathan CS, Black W. Racial and Ethnic Disparities in Early-Stage Lung Cancer Survival. *Chest*. 2017;152(3):587-597. doi:10.1016/j.chest.2017.03.059

9. Varlotto JM, McKie K, Voland RP, et al. The Role of Race and Economic Characteristics in the Presentation and Survival of Patients With Surgically Resected Non-Small Cell Lung Cancer. *Front Oncol*. 2018;8:146. doi:10.3389/fonc.2018.00146

10. Zell JA, Ou S-HI, Ziogas A, Anton-Culver H. Survival improvements for advanced stage nonbronchioloalveolar carcinoma-type nonsmall cell lung cancer cases with ipsilateral intrapulmonary nodules. *Cancer*. 2008;112(1):136-143. doi:10.1002/cncr.23146

11. David EA, Cooke DT, Chen Y, Nijar K, Canter RJ, Cress RD. Does Lymph Node Count Influence Survival in Surgically Resected Non-Small Cell Lung Cancer? *Ann Thorac Surg*. 2017;103(1):226-235. doi:10.1016/j.athoracsur.2016.05.018

12. Saeed AM, Toonkel R, Glassberg MK, et al. The influence of Hispanic ethnicity on nonsmall cell lung cancer histology and patient survival. *Cancer*. 2012;118(18):4495-4501. doi:10.1002/cncr.26686

13. Tantraworasin A, Taioli E, Liu B, Flores RM, Kaufman AJ. The influence of insurance type on stage at presentation, treatment, and survival between Asian American and non-Hispanic White lung cancer patients. *Cancer Med*. 2018;7(5):1612-1629. doi:10.1002/cam4.1331

14. Brzezniak C, Satram-Hoang S, Goertz H-P, et al. Survival and Racial Differences of Non-Small Cell Lung Cancer in the United States Military. *J Gen Intern Med*.

2015;30(10):1406-1412. doi:10.1007/s11606-015-3280-z

15. Du XL, Lin CC, Johnson NJ, Altekruse S. Effects of individual-level socioeconomic factors on racial disparities in cancer treatment and survival. *Cancer*. 2011;117(14):3242-3251. doi:10.1002/cncr.25854

16. Gomez SL, Chang ET, Shema SJ, et al. Survival following Non-Small Cell Lung Cancer among Asian/Pacific Islander, Latina, and Non-Hispanic White Women Who Have Never Smoked. *Cancer Epidemiol Biomarkers Prev*. 2011;20(3):545-554. doi:10.1158/1055-9965.EPI-10-0965

17. Mehta AJ, Stock S, Gray SW, Nerenz DR, Ayanian JZ, Keating NL. Factors contributing to disparities in mortality among patients with non-small-cell lung cancer. *Cancer Med*. 2018;7(11):5832-5842. doi:10.1002/cam4.1796

18. Clément-Duchêne C, Stock S, Xu X, et al. Survival among Never-Smokers with Lung Cancer in the Cancer Care Outcomes Research and Surveillance Study. *Ann Am Thorac Soc*. 2016;13(1):58-66. doi:10.1513/AnnalsATS.201504-241OC

19. David EA, Cooke DT, Chen Y, Perry A, Canter RJ, Cress R. Surgery in high-volume hospitals not commission on cancer accreditation leads to increased cancer-specific survival for early-stage lung cancer. *Am J Surg*. 2015;210(4):643-647. doi:10.1016/j.amjsurg.2015.05.002

20. Smith CB, Bonomi M, Packer S, Wisnivesky JP. Disparities in lung cancer stage, treatment and survival among American Indians and Alaskan Natives. *Lung Cancer*. 2011;72(2):160-164. doi:10.1016/j.lungcan.2010.08.015

21. Wisnivesky JP, McGinn T, Henschke C, Hebert P, Iannuzzi MC, Halm EA. Ethnic Disparities in the Treatment of Stage I Non–Small Cell Lung Cancer. *Am J Respir Crit*

*Care Med*. 2005;171(10):1158-1163. doi:10.1164/rccm.200411-1475OC

22. Aizer AA, Wilhite TJ, Chen M-H, et al. Lack of reduction in racial disparities in cancer-specific mortality over a 20-year period. *Cancer*. 2014;120(10):1532-1539. doi:10.1002/cncr.28617
23. Ellis L, Canchola AJ, Spiegel D, Ladabaum U, Haile R, Gomez SL. Racial and Ethnic Disparities in Cancer Survival: The Contribution of Tumor, Sociodemographic, Institutional, and Neighborhood Characteristics. *J Clin Oncol*. 2018;36(1):25-33. doi:10.1200/JCO.2017.74.2049
24. Jemal A, Ward EM, Johnson CJ, et al. Annual Report to the Nation on the Status of Cancer, 1975–2014, Featuring Survival. *JNCI J Natl Cancer Inst*. 2017;109(9). doi:10.1093/jnci/djx030
25. Lara MS, Brunson A, Wun T, et al. Predictors of survival for younger patients less than 50 years of age with non-small cell lung cancer (NSCLC): A California Cancer Registry analysis. *Lung Cancer*. 2014;85(2):264-269. doi:10.1016/j.lungcan.2014.04.007
26. Clegg LX, Li FP, Hankey BF, Chu K, Edwards BK. Cancer Survival Among US Whites and Minorities. *Arch Intern Med*. 2002;162(17):1985. doi:10.1001/archinte.162.17.1985
27. Jemal A, Clegg LX, Ward E, et al. Annual report to the nation on the status of cancer, 1975-2001, with a special feature regarding survival. *Cancer*. 2004;101(1):3-27. doi:10.1002/cncr.20288
